# Immune and Inflammatory Networks in Myocardial Infarction: Current Research and Its Potential Implications for the Clinic

**DOI:** 10.3390/ijms23095214

**Published:** 2022-05-06

**Authors:** Atsushi Anzai, Seien Ko, Keiichi Fukuda

**Affiliations:** Department of Cardiology, Keio University School of Medicine, Tokyo 160-8582, Japan; kao227@hotmail.com

**Keywords:** myocardial infarction, heart failure, innate immunity, adaptive immunity, immune cells, inflammation, cytokines, chemokines, growth factors, hematopoiesis, clonal hematopoiesis, immunotherapy, clinical trial

## Abstract

Despite recent scientific and technological advances, myocardial infarction (MI) still represents a major global health problem, leading to high morbidity and mortality worldwide. During the post-MI wound healing process, dysregulated immune inflammatory pathways and failure to resolve inflammation are associated with maladaptive left ventricular remodeling, progressive heart failure, and eventually poor outcomes. Given the roles of immune cells in the host response against tissue injury, understanding the involved cellular subsets, sources, and functions is essential for discovering novel therapeutic strategies that preserve the protective immune system and promote optimal healing. This review discusses the cellular effectors and molecular signals across multi-organ systems, which regulate the inflammatory and reparative responses after MI. Additionally, we summarize the recent clinical and preclinical data that propel conceptual revolutions in cardiovascular immunotherapy.

## 1. Introduction

Myocardial infarction (MI) is the most frequent cause of acute sterile cardiac injury. While many therapeutics in current use, including optimal medical therapy and emergent reperfusion strategy, have proven beneficial to improve short-term outcomes in patients with acute MI, post-MI heart failure with large-scale loss of cardiomyocytes is still a global health problem with high long-term mortality [1,2]. Furthermore, although recent epidemiological evidence regarding cardiomyopathy of non-ischemic origin is steadily accumulating, ischemic cardiomyopathy represents a deleterious phenotype leading to poor clinical outcomes [3,4,5], which indicates unmet medical needs, requiring a further understanding of the disease pathophysiology.

MI typically occurs when ruptured or eroded plaque with fresh thrombi disrupts the blood flow of the coronary artery [6,7]. Because the heart requires abundant energy sources and oxygen for its incessant contraction and lacks regenerative capacity, disruption of the coronary blood supply unleashes a cascade of events that irreversibly kills myocardial tissue in the affected regions. Infarct expansion with the necrotic scar triggers progressive left ventricular (LV) dilatation and systolic dysfunction, which eventually leads to heart failure, the malignant syndrome impeding patients’ quality of life and draining healthcare resources, thereby predicting poor long-term outcomes.

Decades of research have uncovered major determinants of maladaptive LV remodeling following MI, of which infarct size can be reduced by primary revascularization therapy and wall stress and elevated neurohumoral factors can be pharmacologically suppressed with β-blockers and angiotensin-converting enzyme inhibitors [8,9]. Another residual determinant whose underlying mechanism is still poorly understood is infarct healing, for which immune and inflammatory activation robustly shapes the complicated process [10,11,12]. Generally, the immune system has evolved to enable host defense against invading pathogens but also to promote tissue repair following sterile tissue injury. However, the immune system may promote prolonged tissue damage, culminating in adverse wound healing due to exaggerated inflammation. Thus, understanding and elucidating the detailed mechanisms underlying immune cell-mediated inflammatory networks aiming towards appropriate post-MI healing process will lead to additional therapeutic approaches to improve patient outcomes.

## 2. Immune Cell Repertoire in the Steady-State Heart

Experimental studies with healthy adult mice, some of which have utilized single-cell RNA sequencing (scRNA-seq), have revealed that diverse leukocyte subsets, including macrophages, lymphocytes, mast cells, and dendritic cells (DCs), reside in the steady-state heart. As in most tissues, the dominant immune cells in the heart are macrophages, which are typically located near the endothelium or within the interstitial space [12,13,14]. For over a decade, there have been fervid discussions and increased interest in the ontogeny and function of tissue-resident macrophages, which argued the foundational concept of the “mononuclear phagocyte system” by Van Furth and colleagues, proposing that blood monocytes continually give rise to tissue macrophages [15]. With the development of sophisticated tools that enable the macrophages in animals to be tracked, we now appreciate that cardiac macrophages are ontogenically diverse and heterogeneous, consisting of several subsets. Details regarding resident cardiac macrophage subsets have been comprehensively reviewed elsewhere [10,16,17,18] and will not be extensively described in this review.

Cardiac resident macrophages may physiologically function in heart development, coronary artery maturation, adaptation to increased tissue strain, and regulation of autonomic innervation. Recent experimental work further identified that resident cardiac macrophages residing in the atrio-ventricular (AV) node electrically couple to cardiomyocytes through connexin-43 containing gap junctions and are critical to maintaining the impulse conduction system [19]. Indeed, macrophage-depleted mice died of fatal bradycardia arrhythmia such as advanced AV block. More recently, Nicolás-Ávila et al. have found that cardiac macrophages actively take up cardiomyocyte-derived exophers that contain defective mitochondria through the phagocytic receptor, Mer tyrosine kinase (MerTK), within the healthy myocardium [20]. Depletion of cardiac macrophages or deficiency of MerTK results in ventricular dysfunction with metabolic abnormalities in the heart. These data suggest that the resident macrophages are indispensable for maintaining cardiac homeostasis.

In addition to macrophages, relatively sparse populations of DCs have been found within cardiac valves, where they presumably monitor and provide surveillance [10]. Mast cells are likely important for triggering immune responses [12]. A small number of B cells and regulatory T cells (Tregs) are also present in cardiac tissue in resting conditions [21,22]. In addition to those immune cells, there are also non-hematopoietic stromal cells such as endothelial cells, pericytes, fibroblasts, and smooth muscle cells in the steady-state heart [13,23,24]. Although stromal cells are likely to be important in part for maintaining tissue integrity, the physiological function of resident immune cells in the steady-state heart, with the exception of resident macrophages, remains largely unknown.

## 3. Immune System in the Wound Healing Process after MI

### 3.1. How Does the Ischemic Heart Initiate the Immune Inflammatory Cascade?

The immune system consists of two major subtypes: innate immunity confers an immediate, non-specific first line of defense against invading pathogens or tissue injury, whereas acquired/adaptive immunity provides a highly specific response that is mainly mediated by lymphocytes [25]. Similar to most organs in the body, the mammalian heart can utilize both the innate and adaptive immune system in order to accommodate not only exogenous pathogens such as bacteria, viruses, and parasites, but also endogenous sterile injury due to, for example, myocardial ischemia [10,12,26].

Once the coronary artery is clogged and myocardial ischemia sustained, parenchymal and cardiomyocyte cell death programs are drastically activated, which result in a sizable amount of cell loss due to apoptosis, necrosis, necroptosis, and/or pyroptosis. Dying cardiomyocytes and other injured cells release endogenous substances such as high mobility group box-1, S100 proteins, interleukin (IL)-1α, heat-shock proteins, mitochondrial DNA, single- or double-stranded RNA, and complement, all of which are known as damage-associated molecular patterns (DAMPs) [10,11,26]. These DAMPs act as danger signals by binding to extracellular or intracellular pattern recognition receptors (PRRs) on surviving immune and stromal cells to initiate the innate immune response.

To date, PRRs are divided into at least four distinct families: Toll-like receptors (TLRs), nucleotide-binding domain leucine-rich repeat containing receptors (also known as Nod-like receptors [NLRs]), retinoic acid-inducible gene-I-like receptors (RLRs), and C-type lectin receptors (CLRs) [27,28]. Among these, TLRs and NLRs have been studied extensively in the context of MI [10,11,26,29], whereas the roles of RLRs and CLRs in the pathophysiology of MI are largely unclear. Downstream signaling resulting from the engagement of TLRs mainly converges on nuclear factor kappa B activation, which robustly produces a wide variety of inflammatory mediators, including inflammatory cytokines, chemokines, growth factors, and cell-adhesion molecules. In animal studies, TLR2 deficiency in hematopoietic cells provides protection against myocardial ischemic injury [30]. The lack of TLR4, which is upregulated in the failing human heart and on circulating monocytes upon ischemic cardiac injury [10], also has protective effects following MI [31], although it is unknown whether this is attributable to the absence of TLR4 activity in hematopoietic or non-hematopoietic cells. NLRs are another key component of a distinct pro-inflammatory mechanism that requires large cytosolic multiprotein complexes called inflammasomes. The NLR family pyrin domain containing 3 (NLRP3) inflammasome serves as a platform for the activation of caspase-1, which is responsible for the production of IL-1β and IL-18 [32,33]. This response requires the induction of pro-IL-1β expression, which is mediated, in part, through the activation of TLRs, indicating the synergy of the TLR and NLR pathways in enhancing the innate immune response. Recent experimental data using bone marrow transplantation revealed that the absence of the NLRP3 inflammasome in hematopoietic cells attenuates adverse cardiac remodeling after MI by reducing tissue damage and promoting proper wound healing [34]. Further studies are needed to elucidate how various PRRs differentially affect immune and non-immune cell subsets during the course of MI, which might represent a novel therapeutic avenue.

### 3.2. Immune Cell Behavior in the Infarcted Heart

Shortly after the onset of innate immune activation, the ischemic heart recruits a diverse repertoire of innate and adaptive immune cells to the injured site. To this end, cardiac endothelial cells become activated to weaken their tight junctions and express adhesion molecules. Furthermore, cardiomyocytes, cardiac fibroblasts, and resident macrophages begin to produce not only inflammatory cytokines, but also chemokines such as C-C motif chemokine ligand 2 (CCL2), CCL7, C-X-C motif chemokine ligand 1 (CXCL1), and CXCL2, which mobilize bone marrow neutrophils and monocytes to the blood in large numbers and also attract the myeloid cells to the infarcted heart from the circulation [35,36,37]. This chemokine-dependent dynamic behavior of myeloid cells is subsequently enhanced by B cells and recruited neutrophils and monocyte/macrophage themselves via secretion of CXCL2, CCL2, and CCL7 [22,35]. Cardiac fibroblasts produce and release granulocyte-macrophage colony-stimulating factor (GM-CSF) in response to the engagement of their PRRs, which not only endows monocytes and macrophages with a pro-inflammatory signature, but also stimulates bone-marrow hematopoiesis [35]. Likewise, various inflammatory mediators such as IL-1β and TLR ligands released from the ischemic myocardium induce the large-scale production of innate immune cells, mainly from hematopoietic stem and progenitor cells (HSPCs) in the bone marrow [38,39,40,41]. Together, these early dramatic events with diverse cellular and molecular changes eventually lead to massive immune cell accumulation in the infarcted heart, which has been reflected by strong clinical evidence demonstrating that leukocytosis occurs after acute MI and is an independent predictor of future cardiovascular events [42,43,44].

Recruited neutrophils and monocytes participate decisively in the cardiac immune inflammatory response. For the first few days following MI, their main functions include scavenging dead and dying cardiomyocytes along with debris. This is an important process involving various scavenger receptors such as MerTK [45]. Monocyte-derived inflammatory macrophages also scavenge DNA fragments released from dying cardiomyocytes through interferon regulatory factor 3 (IRF3) to amplify inflammation [46]. Although, given their short life-span, neutrophil numbers decrease after 3 days and almost entirely disappear after 7 days of MI, they have been shown to improve cardiac healing by modulating macrophage polarization towards the reparative phenotype through the release of neutrophil gelatinase-associated lipocalin [47]. Indeed, antibody-mediated depletion of bulk neutrophils induces cardiac dysfunction and heart failure with increased fibrosis, albeit with no change in infarct size. Neutrophil extracellular traps (NETs) have been recognized as an important mechanism of antimicrobial host defense through a process called NETosis [48]. Although enhanced NET formation likely leads to increased infarct size and progression to heart failure after MI [49,50], whether targeting NETs improves post-MI wound healing process and outcomes remains to be investigated. Recent experimental studies utilizing single-cell transcriptomics analysis identified that lymphocyte antigen 6 complex locus G (Ly6G)-positive neutrophils could be delineated into several distinct clusters [51,52]. Notably, cellular indexing of transcriptomes and epitopes by sequencing analyses revealed that, in contrast with circulating neutrophils, heart-infiltrating neutrophils acquire a unique SiglecF^high^ signature following MI. Although putative pathways mediating neutrophil–macrophage communication in the myocardium have been suggested, whether this specific type of neutrophils is tissue-protective or harmful needs to be elucidated by future research.

There are two main subsets of circulating monocytes in mice, which can be classified by the cell surface marker Ly6C [53,54]. In contrast to Ly6C^low^ monocytes, which adhere to and move along the vascular endothelium to clear damaged cells and trigger inflammatory responses without entering the tissue, Ly6C^high^ monocytes that specifically express chemokine receptor CCR2 continue to accumulate in the ischemic heart, where they abundantly produce inflammatory mediators such as inducible nitrous oxide synthase, reactive oxygen species, interferon (IFN)-γ, tumor necrosis factor (TNF)-α, IL-1, IL-6, and macrophage inflammatory protein 1-α [12,35,38,54,55]. They also release proteases such as matrix metalloproteinases, urokinase-type plasminogen activator, and cathepsins, which digest the pre-existing collagen network and presumably facilitate cell movement. 

Ly6C^high^ monocytes dominate on days 1 to 4 after MI and concurrently differentiate to macrophages with proteolytic and phagocytic activities [12,38,55,56]. King et al. have recently reported that monocyte-derived macrophages scavenge dying cardiomyocyte-derived DNA fragments to activate the cyclic GMP-AMP synthase-stimulator of IFN genes (known as cGAS-STING) signaling pathway [46]. This leads to activation of the transcription factor IRF3, which in turn induces type I IFN production, thereby enhancing infarct inflammation. More detailed profiling with scRNA-seq revealed that the IRF3-dependent IFN response after MI begins in myeloid cell precursors of the bone marrow, which is restrained by Tet methylcytosine dioxygenase 2 (TET2) [57]. Mice lacking Tet2 exhibit increased IFN-stimulated gene induction in the bone marrow myeloid progenitors after MI, consistent with a study showing that hematopoietic loss of Tet2 is associated with adverse cardiac remodeling as a result of activated NLRP3 inflammasome and overproduction of IL-1β in a murine model of MI [58].

Inflammatory macrophages convert their features to the anti-inflammatory phenotype between days 4 and 7 after MI. This biphasic monocyte–macrophage response to ischemic heart injury is similarly observed in human MI [59,60]. The transition is supported partly by intrinsic signals such as nuclear receptor subfamily 4 group A member 1, and defines the reparative response of the post-MI healing process [61]. Reparative macrophages with reduced expression of inflammatory mediators produce IL-10, transforming growth factor (TGF)-β and vascular endothelial growth factor (VEGF), thereby promoting fibrosis and angiogenesis. Cardiac resident macrophages that are self-renewing and independent of monocyte influx also help resolve inflammation and improve adverse remodeling by limiting monocyte recruitment after MI [62,63]. Another study highlighted a subset of macrophages residing in the murine and human pericardial cavity [64]. An overview of this novel macrophage subset is presented below.

A large number of studies have positioned DCs as central regulators of the immune system since their discovery by Ralph Steinman and Zanvil Cohn in 1973 [65]. Their main functions are not only to connect innate and adaptive immunity via antigen presentation through major histocompatibility complex (MHC) class II, but also to secrete various cytokines and growth factors, regulating immune and inflammatory processes [66]. DCs are involved in various types of diseases and MI is not an exception. Bone marrow-derived CD11c^+^ DCs accumulate in the ischemic myocardium, peaking on day 7 post-MI and exhibiting protective effects against adverse cardiac remodeling [67]. Mechanistically, DCs control monocyte/macrophage polarization towards an anti-inflammatory phenotype, thereby leading to favorable wound healing with balanced inflammation. Local injection of tolerogenic DCs has been shown to improve the inflammatory response, wound healing, and cardiac systolic function via activation of reparative macrophages through Tregs in mice [68]. However, a subset of DCs specialized in cross-priming is reportedly pathogenic, likely promoting LV dysfunction via activation of granzyme B-producing CD8^+^ cytotoxic T cells [69]. These data indicate the distinct functional properties of phenotypically distinct DC subsets during the post-MI healing process.

Although the number of T and B lymphocytes that accumulate in the heart is relatively low compared to neutrophils or monocytes/macrophages and peaked around day 7 in a murine model of permanent coronary ligation, those adaptive immune cell subsets do participate in the immune response after MI. The absence of CD4^+^ T cells in CD4-deficient or MHC class II-deficient mice is associated with decreased LV function with inappropriate inflammation [70]. CD4^+^ T cells are activated and proliferate in heart-draining mediastinal lymph nodes where antigen presentation likely occurs in response to MI. Although CD4^+^ T cells can be classified into various subtypes according to their phenotypic markers and functions, the post-MI beneficial function of CD4^+^ T cells is attributed to CD4^+^ Foxp3^+^ Tregs. Indeed, several experimental studies using both genetic and antibody-based approaches to modulate Tregs showed that Tregs are essential for favorable wound healing, scar formation, and inflammation resolution after MI, in part by calibrating macrophage differentiation towards a reparative phenotype [21,71]. In contrast, CD8^+^ T cells [72] and γδT cells [73] seem to promote the inflammatory response and post-MI cardiac dysfunction. The role of T cells in MI has been extensively summarized in recent reviews [74,75]. In addition to T cells, B cells influence post-MI outcomes through various mechanisms. Zouggari et al. reported that B cells are the dominant cellular sources of monocyte-chemoattractant CCL7, thus playing an important role in the activation of inflammatory cascades and development of unfavorable cardiac remodeling [22]. Indeed, anti-CD20 antibody-mediated depletion of B cells limits myocardial injury and improves cardiac function in mice. Importantly, the authors also demonstrated that elevated concentrations of circulating CCL7 or B cell-activating factor belonging to the TNF family are associated with increased risk of cardiovascular death and recurrent MI in humans. These findings are well supported by a recent experimental study identifying that the microRNA 21 (miR21)/hypoxia inducible factor 1 alpha (HIF-1α) pathway in splenic marginal zone B cells is essential for CCL7 production [76]. Additionally, B cells also reside in pericardial adipose tissue where GM-CSF-producing B cells potentially enhance infarct inflammation [77], whereas IL-10-producing CD5^+^ B cells promote the resolution of MI-induced inflammation [78]. These experimental observations related to pericardial B cells are also discussed in the different sections of this review.

Eosinophils, basophils, and innate lymphoid cells (ILCs) are minor populations in the infarcted myocardium; thus, their role in MI has been overlooked for a long time. However, recent extensive research identified their cardioprotective function in maintaining a proper inflammatory response and promoting appropriate tissue healing after MI. Eosinophils develop in the bone marrow under control of the transcription factor GATA binding protein 1 (GATA1) and cytokines IL-3, IL-5, and GM-CSF and have cytoplasmic granules that contain eosinophilic cationic protein, peroxidase, cytokines, and chemokines. Genetic or inducible depletion of eosinophils exacerbates—whereas expansion of eosinophils by systemic administration of IL-5 favorably alters—cardiac function, cell death, and fibrosis [79,80,81]. A key regulator involved is eosinophil-derived IL-4 that potentially activates the reparative macrophage response. Recently, Sicklinger et al. demonstrated that basophils balance the ratio of pro-inflammatory monocytes to anti-inflammatory Ly6C^low^ macrophages via IL-4 and IL-13 in the infarcted heart and therefore lead to the appropriate reparative response [82]. The authors also showed that, in patients with MI, initial low blood basophil counts are associated with worse 1-year clinical outcomes. Another recent research paper reported that ILCs, which are a family of lymphocytes but do not have antigen-specific receptors, expand in the pericardial adipose tissue after experimental MI [83]. Genetic depletion of ILC type 2 (ILC2s) aggravated—whereas low-dose IL-2 treatment-induced ILC2 expansion improved—cardiac function in a mouse model of MI.

**Figure 1:**
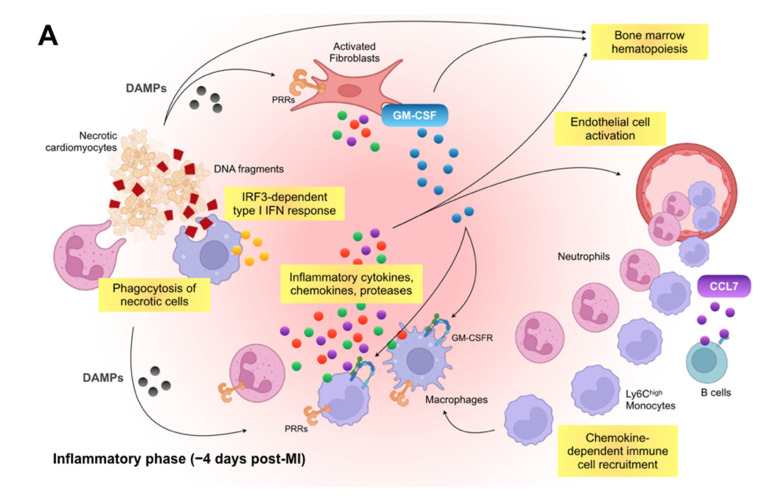

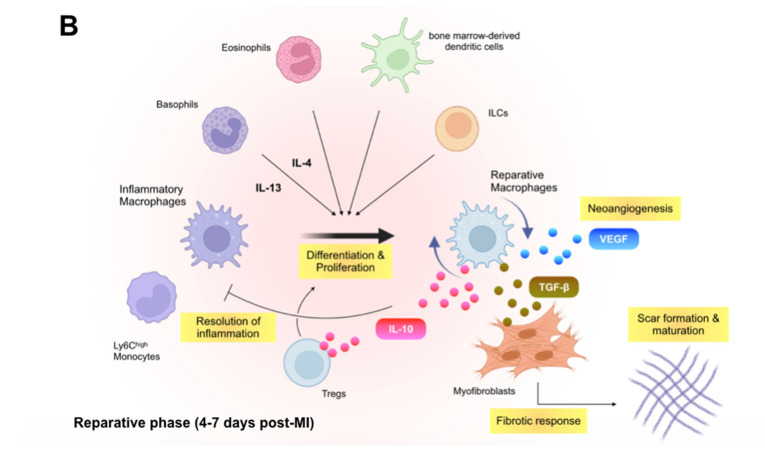
Immune inflammatory response within the infarcted heart. (**A**): Shortly after the onset of myocardial infarction (MI), endogenous damage-associated molecular patterns (DAMPs) released from necrotic cardiomyocytes and other injured cells stimulate surviving resident cells and recruited immune cells to produce inflammatory cytokines, chemokines, and proteases (e.g., interleukin [IL]-1β, C-C motif chemokine ligand 2 [CCL2], and matrix metalloproteinases), which initiate innate immune response. Inflammatory macrophages scavenge dying cardiomyocyte-derived DNA fragments to activate interferon regulatory factor 3 (IRF3)-dependent type I interferon (IFN) signals that enhance infarct inflammation. Cardiac fibroblasts produce and release granulocyte-macrophage colony-stimulating factor (GM-CSF) in response to the engagement of their pattern recognition receptors (PRRs), which not only endows monocytes and macrophages with a pro-inflammatory signature but also stimulates bone marrow hematopoiesis. B cell-derived CCL7 further enhance monocyte accumulation in the infarcted heart. (**B**): In the later phase of ischemic injury, inflammatory macrophages convert their features to the anti-inflammatory phenotype. The transition is supported partly by regulatory T cells (Tregs), basophils, eosinophils, dendritic cells, and innate lymphoid cells (ILCs) and defines the reparative response in the wound healing process. Reparative macrophages as well as Tregs produce anti-inflammatory cytokines (e.g., IL-10) that resolve inflammation. The reparative macrophages likewise produce transforming growth factor (TGF)-β and vascular endothelial growth factor (VEGF), thereby promoting angiogenesis, fibrosis, and scar formation.

Overall, these consecutive events with inflammatory and reparative cascades following MI must be precisely regulated to minimize detrimental tissue damage with the adequate fibrotic response and to avoid the decline of heart function, which in turn leads to optimal outcomes in patients with MI. If the post-MI inflammatory response is exaggerated due to excessive immune cell accumulation, the infarct heals scantly and unavoidably suffers from fragile fibrotic scar. Likewise, if there is a significant lack of robust inflammatory surge, unnecessary debris is inadequately removed, which results in redundant granulation tissue accumulation and an unstable scar, causing fatal complications such as LV rupture in some cases. Figure 1 depicts the possible involvement and function of various immune cells in the post-MI repair process, which consists of several distinct phases. The process starts with intense sterile inflammation with immune cell infiltration (inflammatory phase) followed by resolution of inflammation, myofibroblast proliferation, and neovascularization (reparative phase). After several weeks, immune cell populations in the heart diminish in size towards physiological levels, reparative cells are deactivated, and scar formation with cross-linked extracellular matrix is finally completed in the maturation phase.

### 3.3. Remote Organs Contributing to Immune and Inflammatory Networks after MI

#### 3.3.1. Bone Marrow

Preclinical and clinical data have identified bone marrow as a central organ that produces immune cells and supplies them to the injured heart, thereby essentially contributing to the immune inflammatory response after MI. In bone marrow hematopoiesis, hematopoietic stem cells (HSCs), which are phenotypically characterized by lineage^−^ ckit^+^ Sca1^+^ CD135^−^ CD48^−^ CD150^+^, generate billions of cells every day to maintain homeostatic levels with a tightly controlled proliferation and differentiation process. Upon ischemic cardiac injury, HSC quiescence dissolves followed by massive production of immune cells that subsequently exit the bone marrow, enter the circulation, and reach their destination tissue via adhesion, rolling, and extravasation in response to additional stimuli.

Bone marrow HSCs progressively proliferate within 48 h following cardiac ischemic injury in mice [84]. Heightened bone marrow hematopoiesis is also evidenced in humans as increased bone marrow glucose uptake using 18F-fluorodeoxyglucose (FDG) positron emission tomography (PET), which can be utilized as a surrogate [85]. A more specific subset of HSCs that express CCR2 is located at the top of the hematopoietic hierarchy of emergency myelopoiesis and generates myeloid cells via myeloid translocation gene on chromosome 16 (Mtg16) [84]. Interestingly, Mtg16-deficient mice display decreased levels of systemic monocytes and infarct-associated macrophages, which lead to compromised tissue healing and worse cardiac performance after MI.

The molecular mechanisms by which bone marrow hematopoiesis is stimulated in response to MI partly rely on DAMPs, growth factors, and inflammatory cytokines released from the infarcted myocardium [39,86]. These circulating mediators activate HSPCs harboring the respective receptor in the distant bone marrow to trigger innate immune cell production. For example, cardiac fibroblast-derived GM-CSF stimulates its receptor-positive, myeloid-biased multipotent progenitor 3, one of the distinct subsets of HSPCs, to robustly produce neutrophils and Ly6C^high^ monocytes [35], whereas IL-1β enhances hematopoietic stem cell proliferation by both direct actions on HSCs and through modulation of the bone marrow’s hematopoietic microenvironment after MI [40]. Another key mechanism that drives bone marrow hematopoiesis in response to MI is attributable to increased sympathetic nervous activity, which attenuates HSPC quiescence and dampens retention factors such as CXCL12 in the hematopoietic niche [38,84,87]. Among the sympathetic signals, the β3 adrenergic receptor-dependent pathway on niche cells seems to be critical for immune cell production after MI [84,87].

The hematopoietic niche is a local tissue microenvironment that maintains and regulates stem cells. Recent advances in molecular biology have elucidated that the major cellular components of the bone marrow niche are mesenchymal stromal cells, osteoblasts, and endothelial cells. Although there are outstanding questions concerning the cellular and functional complexities of the niche, the latest experimental studies add to our understanding of the niche’s role in post-MI emergency bone marrow hematopoiesis. For example, adipose tissue-derived leptin regulates quiescence-promoting hematopoietic niche factors in leptin receptor-positive stromal bone marrow cells [88]. Induced deletion of the leptin receptor in the bone marrow niche cells enhances HSPC proliferation and leukocyte production, thereby worsening post-MI myocardial inflammation and outcomes. More recently, it was also revealed that MI provokes endothelial dysfunction, leakage, vascular fibrosis, and angiogenesis in the bone marrow, leading to a detrimental oversupply of inflammatory leukocytes to the injured heart [89]. Mechanistically, VEGFR2, IL-6, and versican, all of which are upregulated in bone marrow endothelial cells in response to MI, are the key regulators for increased hematopoiesis.

#### 3.3.2. Spleen

The spleen is located in the abdomen, directly beneath the diaphragm, and functions as the body’s largest filter that removes older erythrocytes, blood-borne microorganisms, and cellular debris from the circulation [90]. Likewise, the spleen organizes the immune response with a large amount of immune cells such as DCs, macrophages, as well as a large number of lymphocytes, especially B cells, all of which reside within the organ. In addition to these cells, an important study revealed that bona fide undifferentiated monocytes reside in the spleen and outnumber the equivalents in the blood [91]. The reservoir monocytes that assemble in the subcapsular red pulp increase their motility, exit the spleen, and accumulate in injured tissue depending on angiotensin II signaling during the first day of MI. This initial loss of splenic monocytes after MI recovers quickly, as cell numbers in the spleen reach pre-injury levels within 1 week. This recovery is attributed to the generation of monocytes via a process known as extramedullary hematopoiesis, which is partially activated by IL-1β signaling [92]. Intriguingly, splenic HSPCs are derived from the bone marrow and retained by CD169^+^ macrophages via vascular cell adhesion molecule-1 to shape a splenic hematopoietic niche and begin to generate mainly myeloid cells [93]. In humans, increased uptake of 18F-FDG in the spleen revealed by PET imaging has been observed, and suggests splenic hematopoiesis after acute MI [94,95]. While heightened systemic inflammation after MI increases myeloid cell production in the bone marrow and spleen, how the production of immune cells other than neutrophils and monocytes are regulated, and whether it is pathophysiologically important after MI remain to be investigated.

A recent study showed that marginal zone B cells, which are spatially and functionally distinct from follicular B cells, adversely affect post-MI inflammation and LV remodeling [76]. Marginal zone B cell deficiency and marginal zone B cell-specific deletion of miR21 or HIF-1α improve cardiac function via the upregulation of TLR-dependent expression of CCL7 after MI, indicating that the miR21/HIF-1α axis is essential for guiding marginal zone B cell function to mobilize the pro-inflammatory monocytes into the ischemic myocardium.

#### 3.3.3. Pericardial Adipose Tissue and Fluid

Clinically, an increased volume of pericardial adipose tissue is associated with the development of cardiovascular events such as atrial fibrillation, coronary artery calcification, and impaired left atrial and LV structure and function. However, the concrete mechanisms underlying how fat that is anatomically close to the myocardium and cardiac vasculature impacts cardiovascular disease (CVD) have not been fully investigated. A recent study showed that large lymphoid clusters contain a substantial amount of T and B cells within the pericardial adipose tissue [77]. In particular, pericardial B cells are activated with marked expansion and a variety of functions that influence the outcome after MI. A subset of pericardial B cells proliferates and produces GM-CSF in response to MI, which lead to DC activation followed by IL-17 secretion by T cells. These responses trigger granulopoiesis through granulocyte colony-stimulating factor production and consequently promote the decline of heart function. Although the study did not provide data with conditional knockout mice lacking GM-CSF specifically in B cells, it was the first study proposing the detrimental function of pericardial adipose tissue B cells in MI. Another subset of B cells that expresses CD5 and produces IL-10 is also enriched in the pericardial adipose tissue via IL-33 and CXCL13 [78]. B cell-specific deletion of IL-10 worsens cardiac function, exacerbates myocardial injury, and delays the resolution of inflammation following acute MI. These data suggest that phenotypically and functionally distinct B cell subsets in the pericardial adipose tissue have differential roles in post-MI pathophysiology.

In addition to lymphocytes, ILCs also reside in the pericardial fat and expand in response to MI. ILC2s seem to be protective for proper tissue healing response, as the absence of ILC2s increases the accumulation of inflammatory monocytes and macrophages and negatively impacts LV function after MI in mice [83]. Exogenous infusion of low-dose IL-2 that significantly expands the ILC2 population improves cardiac function not only in mice but also in humans, suggesting that administration of the cytokine could be a novel therapeutic strategy for avoiding progression to heart failure in patients with MI.

A recent study suggested that pericardial fluid contains a discrete subset of macrophages characterized by GATA6 expression similar to macrophages in pleural and peritoneal spaces [64]. Transcriptional profiling of these GATA6-expressing pericardial cavity macrophages displayed the enriched expression of genes related to protein and nucleic acid metabolism, which are unique relative to macrophages in the neighboring heart, albeit with more similar RNA expression profiles among the different serosal cavities. Following experimental MI, pericardial cavity macrophages are recruited to the remote, non-infarcted myocardium between days 3 and 7 independent of circulating monocyte infiltration. Although there is no significant decline in LV systolic function compared to controls, loss of this specialized macrophage population enhances interstitial fibrosis and cardiac stiffness, leading to diastolic dysfunction after ischemic injury. Notably, Gata6^+^ macrophages are also present in human pericardial fluid, suggesting the important immunomodulatory role of this distinct reparative cell subset in human heart disease. 

#### 3.3.4. Mediastinal Lymph Node

Mediastinal lymph nodes are located close to the heart in the mediastinal space and garner a lot of adaptive immune cells. Hofmann et al. injected fluorescent microparticles into the anterior LV free wall in mice subjected to MI surgery and found that these labeled microparticles were phagocytosed in CD11b^+^ putative antigen-presenting cells in right upper mediastinal but not in cervical or inguinal lymph nodes, spleen, or bone marrow, suggesting that mediastinal lymph nodes are heart-draining lymph nodes, where antigen presentation likely occurs in the context of MI [70]. Indeed, OT-II transgenic mice show negligible T cell activation, and these mediastinal lymph nodes in wild-type mice are consistently enlarged with increased T cell numbers in response to MI, but not after sham surgery.

Although CD4^+^ T cells can be subdivided into several subpopulations according to their cell surface, intracellular, and nuclear markers, the detrimental phenotype in CD4-deficient mice subjected to MI is likely attributed to the lack of Tregs. In the absence of Tregs, macrophage function shifts towards the inflammatory phenotype, whereas activation of Tregs promotes the reparative function of cardiac macrophages to produce arginase-1, IL-13, osteopontin, and TGF-β, which modulate the fibrotic response, leading to favorable wound healing after MI [21]. Consistently, in vivo expansion of Tregs by means of either the adoptive transfer of Tregs or a CD28-superagonistic antibody attenuated myocardial pro-inflammatory cytokine expression and immune cell infiltration in a rodent MI model [96]. These data indicated that heart-draining mediastinal lymph nodes are essential places for endogenous lymphocytes, especially Tregs, which expand with antigen presentation to regulate repair of the ischemic myocardium.

**Figure 2:**
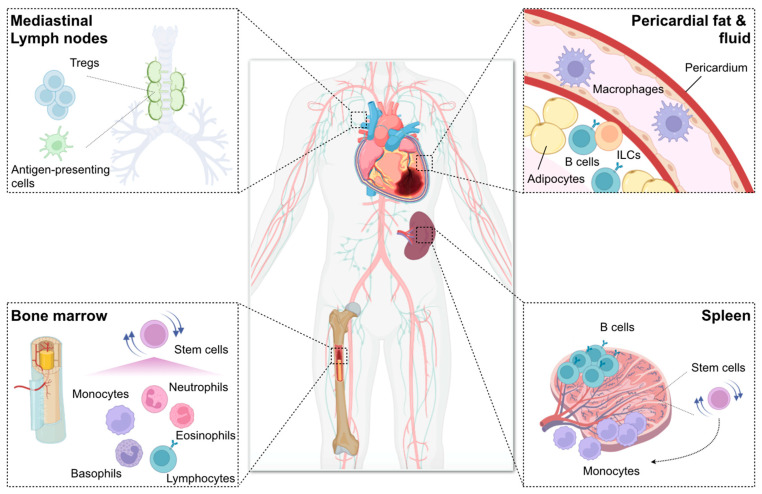
Remote organs contributing to immune and inflammatory networks after myocardial infarction (MI). Bone marrow generates billions of immune cells and supplies them to the injured heart, thereby essentially contributing to the immune inflammatory response after MI. Infarct-derived inflammatory cytokines (e.g., interleukin [IL]-1β), damage-associated molecular patterns (e.g., S100A8), and growth factors (e.g., granulocyte-macrophage colony-stimulating factor [GM-CSF]) as well as increased sympathetic nervous signals stimulate bone marrow hematopoietic stem cell proliferation and immune cell production. Spleen also has stem cells and produces mainly myeloid cells in a process known as extramedullary hematopoiesis. Splenic reservoir monocytes exit the spleen and accumulate in injured tissue depending on angiotensin II signaling during the first day of MI. The miR21/HIF-1α axis in splenic marginal zone B cells is essential for C-C motif chemokine ligand 7 production, thereby promoting inflammatory monocyte mobilization to the infarcted heart. B cells also reside in pericardial adipose tissue where GM-CSF-producing B cells potentially enhance infarct inflammation, whereas IL-10-producing CD5^+^ B cells promote the resolution of MI-induced inflammation. Innate lymphoid cells (ILCs) also expand in the pericardial adipose tissue after MI and exhibit beneficial function. Pericardial fluid contains a discrete subset of macrophages expressing GATA binding protein 6, which may be protective against diastolic heart failure following MI. Mediastinal lymph nodes are heart-draining lymph nodes where regulatory T cells (Tregs) expand and proliferate with antigen presentation to regulate repair of the ischemic myocardium.

Collectively, multiple organs outside the injured heart are important underpinnings, where innate and adaptive immune cells expand, differentiate, repopulate, and gain a variety of functions. As a result, immune cells in the remote organs essentially contribute to the cardiac wound healing process following MI by secreted mediators or direct infiltration to the heart (Figure 2).

## 4. Clonal Hematopoiesis: An Emerging Protagonist Promoting CVD

As we age, somatic mutations accumulate in bone marrow HSPCs, some of which confer a competitive advantage or fitness, leading to clonal expansion of the mutated cells. Consequently, the bone marrow expels clonal descendants of the mutant stem cells into peripheral circulation, resulting in more than 10% of individuals by age 70 with these clones accounting, on average, for up to 20% of their peripheral leukocytes. The presence of such clones, referred to as clonal hematopoiesis, indicates a premalignant condition capable of developing into hematologic cancers such as acute myeloid leukemia and myelodysplastic syndromes, albeit with a rare progression rate. Although there has been little information regarding the role of age-related clonal hematopoiesis on chronic disorders, recent epidemiological studies with exome sequence analyses showed that clonal hematopoiesis is associated with an approximately 40% increased cardiovascular risk, independent of traditional risk factors [97,98]. The incidence of cardiovascular events likely depends on the clone size: a clone size of more than 20% of blood cells results in a higher coronary calcium score, which is a surrogate for atherosclerotic burden detected by computed tomography, whereas a smaller clone size confers a relatively lower risk for developing CVD.

Genes frequently mutated in the expanded cells are DNA methyltransferase 3 alpha (DNMT3A), TET2, addition of sex combs such as one (known as ASXL1), Janus kinase 2 (known as JAK2), and tumor protein p53 (known as TP53) and the odds ratio for developing CVD increases in the presence of these somatic mutations. The driver gene mutations often affect epigenetic regulation, thereby altering cell proliferation and phenotype. For example, the TET2 gene encodes an enzyme that catalyzes DNA hydroxymethylation; therefore, its mutation confers both increased proliferation capacity and inflammatory signature to the mutated cells. A series of experiments using Tet2-deficient mice was performed to uncover the underlying mechanisms by which TET2 mutation leads to poor cardiovascular outcomes. In a mouse model of atherosclerosis, hematopoietic or myeloid cell-specific loss of Tet2 accelerates plaque expansion and the inflammatory response mainly due to phenotypic alteration of aortic macrophages, exhibiting increased NLRP3 inflammasome-dependent production of IL-1β. Interestingly, while circulating leukocyte counts remain unchanged, Tet2-deficient monocytes dominate in the blood over time [98,99].

**Figure 3:**
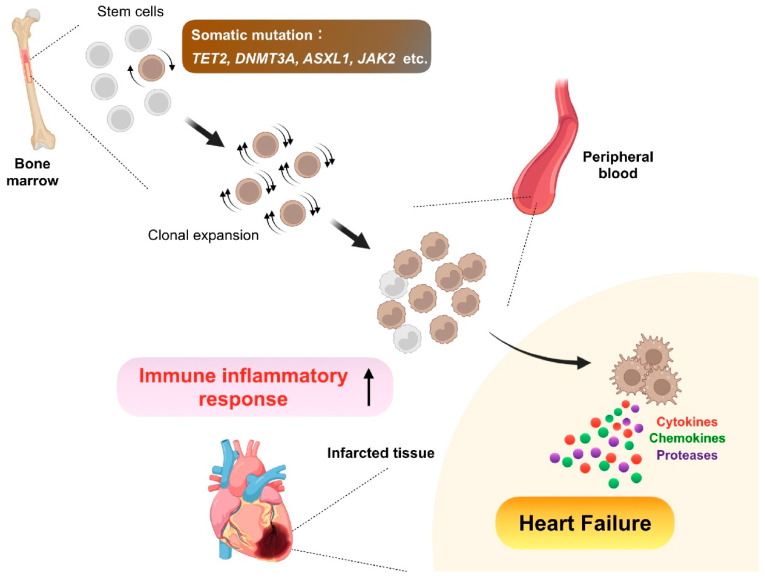
Clonal hematopoiesis. As we age, somatic mutations accumulate in bone marrow hematopoietic stem cells, some of which confer a competitive advantage or fitness, leading to clonal expansion of the mutated cells. Consequently, the bone marrow expels clonal descendants of the mutant stem cells into peripheral circulation. In this premalignant condition called clonal hematopoiesis, genes frequently mutated are Tet methylcytosine dioxygenase 2 (TET2), DNA methyltransferase 3 alpha (DNMT3A), addition of sex combs like 1 (ASXL1), and Janus kinase 2 (JAK2), and the somatic mutations confer both increased proliferation capacity and inflammatory signature to the mutated cells, thereby leading to adverse post-infarction heart failure with enhanced inflammation.

In the context of post-MI heart failure (Figure 3), Tet2 deficiency likewise impacts maladaptive cardiac remodeling after MI. Experimentally, a competitive bone marrow transplantation model with Tet2-deficient cells that mimic TET2 mutation-driven clonal hematopoiesis in humans or myeloid cell-specific ablation of Tet2 showed worsened cardiac remodeling and function in a murine model of permanent coronary ligation, in combination with increased inflammatory response characterized by elevated IL-1β expression [58]. Additionally, deep sequencing analysis of bone marrow-derived mononuclear cells retrieved from patients with ischemic heart failure recently indicated that hematopoietic mutations in not only TET2 but also the most commonly mutated driver gene DNMT3A may be significantly associated with disease progression and a poor prognosis [100]. Although the sample size was small, the composite outcomes, including death from CVD and hospitalization due to heart failure, were positively correlated with the size of mutant clones in the study. The impact of DNMT3A mutation on immune cells such as circulating monocytes and T cells has also been reported [101].

Clinical and preclinical data further indicated that the presence of clonal hematopoiesis relates to adverse outcomes in various forms of CVD [102,103,104,105]. Likewise, novel mechanistic insights associated with mutations of clonal hematopoiesis-related driver genes other than TET2 and DNMT3A have emerged [106,107,108,109]. There have also been comprehensive reviews regarding the contribution of clonal hematopoiesis to the incidence and development of CVD [110,111,112,113,114,115]. Since the association of CVD with clonal hematopoiesis is a recent discovery, many open questions remain to be answered. First, although clonal hematopoiesis is associated with age, male sex, ethnicity, smoking, and type 2 diabetes, it is not fully known how we acquire somatic mutations that lead to uncontrollable stem cell expansion and adverse clinical outcomes. Second, whether clonal hematopoiesis affects immune cells other than myeloid cells and T cells that promote CVD remains to be investigated. Third, as-yet-unknown somatic mutations might carry cardiovascular risks. Finally, whether targeting specific inflammatory signals is valuable for precision medicine in patients harboring specific mutations encoding for the driver genes is one of the important clinical questions, which must be elucidated by future studies.

## 5. Potential Therapy Targeting the Immune Inflammatory Pathways after MI

As discussed above, numerous experimental and clinical publications have described the relationship between the immune inflammatory response and post-MI cardiac wound healing process. Although there is a significant lack of direct evidence from well-controlled clinical studies with a sufficient sample size which show that the modulation of immune inflammatory signals can improve clinical outcomes after MI, we are now entering a new era in which the gap between experimental observations and clinical practice should be lessened by clinical investigations.

The recent Canakinumab Anti-inflammatory Thrombosis Outcomes Study (CANTOS) was the first clinical trial, demonstrating that inflammation in fact drives CVD and targeting inflammation could reduce cardiovascular risk [116]. Patients with a history of MI and high serum C-reactive protein (CRP) levels, who received a monoclonal antibody against IL-1β, canakinumab, had a significantly lower incidence of recurrent MI, stroke, or death from CVD compared to patients receiving placebo. However, there was a small but statistically significant increase in the incidence of infections in those receiving canakinumab. These findings indicate that anti-inflammatory therapy could be a double-edged sword, and its application in optimizing the benefit-risk ratio should be warranted.

One of the other anti-inflammatory agents that has garnered attention is colchicine, which is generally used for the treatment of gout, pericarditis, and familial Mediterranean fever. Colchicine is multifunctional, and its main mechanism of action is regulating NLRP3 inflammasome activity, thereby inhibiting the release of the pro-inflammatory cytokines IL-1β and IL-18. Colchicine’s therapeutic effects continue for several days after administration, even at low doses, because it concentrates in the target cells where the molecule remains active. The low-dose colchicine (LoDoCo) study followed by two large clinical trials, the Colchicine Cardiovascular Outcomes Trial (COLCOT) and LoDoCo2, exhibited a favorable impact of colchicine on cardiovascular outcomes [117,118], although colchicine treatment was associated with an increased incidence of infections, as seen in the CANTOS trial. Colchicine reduces infarct size and inflammatory parameters after acute MI [119], but there have also been conflicting results [120]. The effect of colchicine in combination with spironolactone in patients with ST-elevation MI (STEMI) receiving percutaneous coronary intervention with SYNERGY bio-absorbable polymer drug eluting stent on major adverse cardiovascular events including heart failure is under evaluation in the CLEAR-SYNERGY registry. Collectively, the results from upcoming studies will inform how colchicine can be used in various cardiovascular settings. 

Relatively smaller clinical trials with the IL-1 receptor antagonist, anakinra, demonstrated its anti-inflammatory properties in patients with MI. For example, The MRC-ILA-HEART study enrolled patients with non-STEMI and showed a decrease in the area under the curve of the inflammatory biomarker CRP [121]. Likewise, Virginia Commonwealth University Anakinra Remodeling Trial 3 (VCUART3) demonstrated attenuation of systemic inflammatory response in patients with STEMI treated with anakinra [122]. Importantly, anakinra also reduced the incidence of death, new-onset heart failure, or heart failure hospitalization versus placebo, albeit with no significant difference in LV geometry and in the incidence of serious infections.

Beyond IL-1 and colchicine, other targets involved in immune inflammatory response should be considered. Several preclinical studies have revealed the detrimental role of IL-6 receptor signaling in MI [123,124] and agents that neutralize the signaling are readily available in the clinic. Although tocilizumab that blocks the IL-6 receptor (IL-6R) causes increased levels of triglycerides and therefore may not be suitable for treating chronic CVD, studies on the short-term administration of the IL-6R antagonist during acute MI have yielded encouraging results. The ASSAIL-MI trial is a randomized, double-blind, placebo-controlled trial that evaluated the effect of tocilizumab on myocardial salvage in acute STEMI [125]. Intriguingly, although the study was not powered to show an effect on clinical outcomes [126,127], a single infusion of tocilizumab promptly after the onset of MI increased myocardial salvage based on magnetic resonance imaging [125].

The LILACS trial was designed to evaluate the safety and pharmacodynamics of low-dose IL-2 in patients with stable ischemic heart disease or acute coronary syndrome [128]. The concept of this trial was based on the strong experimental observation showing that IL-2 plays a key role in the development, expansion, and survival of Tregs, which consistently exhibit protection against CVD. Using low-dose aldesleukin (a recombinant human IL-2) administration, which skews the T cell balance towards Tregs and may also possibly induces ILC2 expansion [83] (see above), the phase I/II trial showed its safety and tolerability in clinical use, awaiting the future results from the ongoing phase III trial. Although other mediators, including IL-5, IL-10, IL-17, IL-23, and GM-CSF, may also be valuable to target based on experimental findings, to date, there is no available information regarding clinical studies testing the effect of targeting those inflammatory cytokines and growth factors in patients with MI.

As a cell-based approach, RITA-MI, a prospective, open-label, phase I/IIa trial, examined the pharmacological effect of rituximab in patients with STEMI [129]. Rituximab is a monoclonal antibody that targets human B cells and is generally used for the treatment of autoimmune disease and cancers. The concept of this trial likewise stems from strong preclinical work, as discussed above [22]. A single intravenous injection of rituximab achieved more than 90% depletion of circulating B cells within 30 min and appeared safe without affecting immunoglobulin levels. To further investigate both safety and the ideal dose, a multinational, randomized, double-blind, placebo-controlled phase IIb trial called RITA-MI 2 is ongoing to assess the impact of B cell depletion using rituximab on LV dysfunction and cardiac remodeling after acute MI. Selected clinical studies targeting immune inflammatory pathways in patients with MI are listed in Table 1. Additionally, recent in silico theranostic technologies identified ovatodiolide, a bioactive compound isolated from *Anisomeles indica* (L.) Kuntze, as a potential novel therapeutic option to target cytokine–cytokine receptor interactions, chemokine signaling, immune and inflammatory responses, and metabolic dysregulation in MI [130], which needs to be validated in future clinical and experimental research.

## 6. Concluding Remarks

The ultimate goal of understanding how the immune system is involved in multi-organ and multi-cellular networks that govern inflammatory and reparative programs following MI is the development of innovative therapeutic strategies that promote optimal tissue healing and improve patient outcomes. In the past few decades, there have been difficulties translating experimental findings to clinical practice. However, recent revolutions in immune cell profiling, cell tracing, and sequencing technologies have deepened our knowledge of immune cell behavior in physiological and pathological conditions and provided a way forward. Large-scale outcomes-based clinical studies that substantiate the approach of selectively suppressing excessive inflammation while preserving reparative functions are needed in the near future.

## Figures and Tables

**Table 1:** Selected clinical studies targeting immune inflammatory pathways in myocardial infarction.

Trial	Conditions	Interventions	Outcomes	Ref.
ASSAIL-MI	First-time STEMI	Single dose of tocilizumab (IL-6R antibody) vs. placebo	Improved myocardial salvage with tocilizumab	NCT03004703 [125]
CLEAR-SYNERGY	STEMI treated with primary PCI	SYNERGY stent plus colchicine and spironolactone or placebo	Ongoing	NCT03048825
COVERT-MI	First STEMI treated with primary PCI	5-day administration of colchicine vs. placebo	Colchicine did not reduce infarct size	NCT03156816 [120]
LILACS	Stable CAD and ACS	Low-dose aldesleukin (recombinant IL-2)	This phase I/II trial showed safety and tolerability of aldesleukin	NCT03113773 [128]
RITA-MI	Acute anterior STEMI treated with primary PCI	Single intravenous injection of rituximab (anti-CD20 monoclonal antibody)	This phase I/IIa trial showed safety and tolerability of rituximab	NCT03072199 [129]
VCUART3	Acute STEMI	Anakinra (IL-1R antagonist) vs. placebo	Anakinra significantly reduced systemic inflammatory response	NCT01950299 [122]
VCUART4	Acute STEMI	Anakinra (IL-1R antagonist) vs. placebo	Not yet recruiting. Planned to assess how well anakinra can protect participants from developing heart failure	NCT05177822
